# A Glacier Bacterium Produces High Yield of Cryoprotective Exopolysaccharide

**DOI:** 10.3389/fmicb.2019.03096

**Published:** 2020-02-11

**Authors:** Pervaiz Ali, Aamer Ali Shah, Fariha Hasan, Norbert Hertkorn, Michael Gonsior, Wasim Sajjad, Feng Chen

**Affiliations:** ^1^Institute of Marine and Environmental Technology, University of Maryland Center for Environmental Science, Baltimore, MD, United States; ^2^Applied Environmental and Geomicrobiology Laboratory, Department of Microbiology, Quaid-i-Azam University, Islamabad, Pakistan; ^3^Research Unit Analytical Biogeochemistry, Helmholtz Zentrum München, Munich, Germany; ^4^Chesapeake Biological Laboratory, University of Maryland Center for Environmental Science, Baltimore, MD, United States; ^5^Department of Biological Sciences, National University of Medical Sciences, Rawalpindi, Pakistan

**Keywords:** Karakoram, psychrotrophs, exopolysaccharide, glacier bacteria, cryopreservation

## Abstract

*Pseudomonas* sp. BGI-2 is a psychrotrophic bacterium isolated from the ice sample collected from Batura glacier, Pakistan. This strain produces highly viscous colonies on agar media supplemented with glucose. In this study, we have optimized growth and production of exopolysaccharide (EPS) by the cold-adapted *Pseudomonas* sp. BGI-2 using different nutritional and environmental conditions. *Pseudomonas* sp. BGI-2 is able to grow in a wide range of temperatures (4–35°C), pH (5–11), and salt concentrations (1–5%). Carbon utilization for growth and EPS production was extensively studied and we found that glucose, galactose, mannose, mannitol, and glycerol are the preferable carbon sources. The strain is also able to use sugar waste molasses as a growth substrate, an alternative for the relatively expensive sugars for large scale EPS production. Maximum EPS production was observed at 15°C, pH 6, NaCl (10 g L^–1^), glucose as carbon source (100 g L^–1^), yeast extract as nitrogen source (10 g L^–1^), and glucose/yeast extract ratio (10/1). Under optimized conditions, EPS production was 2.01 g L^–1^, which is relatively high for a *Pseudomonas* species compared to previous studies using the same method for quantification. High-performance anion-exchange chromatography with pulsed amperometric detection (HPAEC-PAD) analysis of EPS revealed glucose, galactose, and glucosamine as the main sugar monomers. Membrane protection assay using human RBCs revealed significant reduction in cell lysis (∼50%) in the presence of EPS, suggesting its role in membrane protection. The EPS (5%) also conferred significant cryoprotection for a mesophilic *Escherichia coli* k12 which was comparable to glycerol (20%). Also, improvement in lipid peroxidation inhibition (*in vitro*) resulted when lipids from the *E. coli* was pretreated with EPS. Increased EPS production at low temperatures, freeze thaw tolerance of the EPS producing strain, and increased survivability of *E. coli* in the presence of EPS as cryoprotective agent supports the hypothesis that EPS production is a strategy for survival in extremely cold environments such as the glacier ice.

## Introduction

Low temperature is very common among extreme environments on the earth. About 85% of the earth’s biosphere is permanently exposed to temperatures below 5°C and glaciers account for 10% of it (Margesin and Miteva, 2011). The cold habitats have been successfully colonized by microorganisms which survive and even grow at temperatures near freezing point of water. The cold-adapted organisms are called psychrophiles or psychrotrophs with the former isolated from permanently cold environments and the latter tends to dominate environments that undergo thermal fluctuations (Russell, 1990). Cold-adapted microorganisms have evolved unique mechanisms to cope with the challenges. These adaptations include increased membrane fluidity through changes in the lipid profile of the membrane (Russell, 1997; Chintalapati et al., 2004; Králová, 2017; Siliakus et al., 2017), conformational flexibility, and increased enzyme activity involved in key cellular processes such as transcription and translation (Lim et al., 2000; Russell, 2000; Ernst et al., 2018), induction of cold-shock proteins (CSPs) (Phadtare, 2004), production of antifreeze proteins (AFPs) (Gilbert et al., 2004, 2005; Lorv et al., 2014), and production of cryoprotectants such as exopolysaccharides (EPSs; Krembs et al., 2002; Aslam et al., 2012; Carrion et al., 2015).

Exopolysaccharides are extracellular polysaccharide polymers which are produced and secreted outside the cell by microorganisms (Sutherland, 1972). Microorganisms produce polysaccharide in two forms, EPS and capsular polysaccharides (CPS). The EPS or slime either remains loosely attached to the cells or is completely released into the surrounding environment. The CPS remains strongly associated to the cell envelope through covalent bond and plays role in pathogenesis. Many microorganisms including bacteria (Sardari et al., 2017), archaea (Squillaci et al., 2016), cyanobacteria (Bemal and Anil, 2017), fungi (Sun et al., 2016), yeast (Han et al., 2018), and microalgae (García-Cubero et al., 2018) are known to produce EPSs. EPS provides protection against the predators, antimicrobial agents and assist microorganisms to endure extremes of temperature (Casillo et al., 2017), salinity (Bemal and Anil, 2017; Isfahani et al., 2018), and desiccation (Tamaru et al., 2005; Knowles and Castenholz, 2008). EPSs are also essential for the attachment of microorganisms to other surfaces which may be biotic or abiotic (Janczarek et al., 2015), nutrient uptake (Kalpana et al., 2018), and most importantly in biofilm formation (Limoli et al., 2015; Rossi and De Philippis, 2015).

Exopolysaccharide producing microorganisms have been isolated from diverse extreme environments including marine hot springs and deep sea hydrothermal vents (Arena et al., 2009; Panosyan et al., 2018), polar and cold marine environments (Mancuso Nichols et al., 2004; Aslam et al., 2012; Liu et al., 2013; Casillo et al., 2017), and saline and hypersaline environments (Poli et al., 2007; Joulak et al., 2019).

*Pseudomonas* species have been reported previously for the production of EPSs. These EPSs demonstrated a variety of functional activities ranging from metal removal (Maalej et al., 2015; Sathiyanarayanan et al., 2016), gelling and emulsification activity (Carrion et al., 2015; Maalej et al., 2016), biofilm formation (Jennings et al., 2015), antioxidant activity (Sirajunnisa et al., 2016), and antibacterial activity (Maalej et al., 2017). Analysis of monosaccharide units is a critical way for structural characterization of the EPSs. EPSs with only a single type of monomeric unit are called homopolysaccharides and with more than one type of sugar monomers unit are termed as heteropolysaccharides. Monosaccharide composition of the EPSs produced by members of the *Pseudomonas* species represent diverse chemical structures. The EPS from *Pseudomonas stutzeri* AS22 was a heteropolysaccharide consisting of glucose, mannose, and lactyl rhamnose (Maalej et al., 2014). Sathiyanarayanan et al. (2016) reported a heteropolysaccharide from the psychrotrophic *Pseudomonas* sp. PAMC 28620, with glucose, galactose, fucose, mannose, rhamnose, and ribose as monomer units. Antarctic bacterium, *Pseudomonas* sp. ID1 produced heteropolysaccharide comprised of glucose, galactose, and fucose sugar monomers (Carrion et al., 2015). *P. stutzeri* XP1 produced dextran, a homopolysaccharide consisting of glucose units (Zhao et al., 2018).

In recent years, rapid depletion of natural resources and increased demand for natural polymers for pharmaceutical, food, and other industrial applications has led to a remarkable interest in polysaccharides produced by microorganisms. Bacteria produce diverse structural and functional EPSs that could play an important role in biotechnology and industry. Xanthan and Gellan are two of the well-known commercially available bacterial EPS with generally regarded as safe (GRAS) status. Xanthan is used in foods, petroleum industry, pharmaceuticals, cosmetics, personal care products, and agriculture (Sutherland, 2001; RehmEd., 2009; ImesonEd., 2011; YangEd., 2011). Gellan has a wide application in foods, pet food, pharmaceuticals, and research (agar substitute and gel electrophoresis) (Sutherland, 2001; Fialho et al., 2008; RehmEd., 2009; ImesonEd., 2011; Prajapati et al., 2013). Dextran, alginate, curdlan, and cellulose are other bacterial EPS that have industrial and medical relevance with significant commercial value. Some EPSs have been reported for anticancer, antiviral, antibacterial, antiulcer, antioxidant, and immunomodulation activities (Laroche and Michaud, 2007; Arena et al., 2009; Novak and Vetvicka, 2009; Mahdhi et al., 2017; Zhu et al., 2018; Zhang et al., 2019). This is why the beneficial effects of probiotics to human health are partly attributed to EPSs produced by bacteria.

Nature is the best reservoir and less explored extreme environments can be promising sources of microbial metabolites of industrial relevance. In contrast to other extreme environments, little has been reported on EPS producing bacteria from the cold environments, particularly from glaciers of the non-polar regions. Hence, there is a continuous interest for bioprospecting of the cold environments such as the glaciers as they are the least explored niche. Glaciers are considered harsh environments of the biosphere harboring a special biotic community of psychrophilic and psychrotrophic organisms. The multidisciplinary study of proglacial and subglacial ecosystems is only in its infancy and the literature is rapidly expanding as a result of the potential role of microorganisms inhabiting these environments. A great number of glaciers outside Polar Regions are located in the Karakoram Mountains, which also includes Batura glacier. There are very limited reports available on the diversity and biotechnological potential associated with bacteria inhabiting the Karakoram glaciers.

The Himalaya-Karakoram-Hindukush (HKH) is the most glaciated area outside of the Polar Regions and is therefore considered the third pole of our planet (Zhang et al., 2015). These ranges meet at a junction point in the northern part of Pakistan where they host at least 5000 glaciers in its geographical boundary and serve fresh water to a large portion of their population (Rasul et al., 2011). These glaciers are less explored compared to the polar glaciers in terms of its bacterial diversity and functionality. Batura glacier, with a latitude 36°32′N and longitude 74°40′E, is one of the longest non-polar glaciers in the world. We explored bacterial diversity of this glacier using culture-dependent and culture-independent methods. The glacier is dominated by a diverse group of psychrotrophic bacteria (unpublished work). *Pseudomonas* sp., BGI-2 was used for detailed study among other bacteria due to its high abundance in the glacier ice sample and rapid growth at low temperatures. *Pseudomonas* sp. BGI-2 produced highest amounts of EPS among the seven EPS producing isolates from the glacier samples. Previously, draft genome sequence data revealed that BGI-2 genome has 11 EPS-producing genes compared to none in the seven closely related mesophilic *Pseudomonas* strains (Ali et al., 2019). We also found more stress response genes in the genome of BGI-2 than in their closely related mesophilic counterparts. BG1-2 is able to thrive in the glacier likely due to its resilience to cope with the harsh glacier conditions which includes frequent freeze-thaw cycles, high UV exposure, desiccation, and low nutrient availability. This work demonstrates that EPS produced by a cold-adapted *Pseudomonas* sp. BGI-2 provides protection against harsh environmental conditions (freeze thaw events) and chemical lysis (detergents). The EPS produced by this strain conferred significant cryoprotection for a mesophilic *Escherichia coli* k12, suggesting its role in cryoprotection. Also, the EPS provided significant stability to the membranes of RBCs subjected to detergent lysis. Increased inhibition of lipid peroxidation in the presence of EPS further validated its role in membrane protection. As most of the previous works on EPSs have focused on its use as gelling or emulsifying agent in food industry, this work will pave new avenues for the EPS such as its use as a cryoprotective and membrane stabilizing agents. Particularly its application in the cryopreservation industry will be a significant milestone as the cryoprotective agents currently in use [most commonly dimethyl sulfoxide (DMSO) and methanol] are toxic to cells at higher concentrations.

## Materials and Methods

### Sample Collection and Bacterial Isolation

Samples were collected on December 24 in the year 2015. An ice axe wiped with 70% ethanol was used for drilling and sampling of the ice. Surface ice up to 5 inches was removed, discarded, and the underlying ice was collected aseptically into sterile 500 mL polypropylene wide-mouth sample bottles. Handling of the samples was performed according to standard microbiological techniques to avoid any contamination. The tubes were sealed, placed in an isothermal box, and transported to Microbiology Research Laboratory, Quaid-i-Azam University, Islamabad, and stored at −20°C.

For bacterial isolation, two different approaches were used: (a) direct plating of the samples on R2A, TSA, and LB agar and (b) enrichment of the sample in R2A broth (Difco), tryptic soya broth (Oxoid), and Luria-Bertani broth (Miller) before plating on agar plates. For direct plating, different dilutions of the sample were plated on agar plates using spread plate method. Plates were incubated at 4 and 15°C and observed daily for colony appearance. For sample enrichment, 10 mL of melted ice sample was added in 100 mL of R2A broth (yeast extract 0.5 g L^–1^, protease peptone 0.5 g L^–1^, casamino acids 0.5 g L^–1^, dextrose 0.5 g L^–1^, soluble starch 0.5 g L^–1^, sodium pyruvate 0.3 g L^–1^, dipottasium phosphate 0.3 g L^–1^, magnesium sulfate 0.05 g L^–1^), tryptic soy broth (pancreatic digest of casein 15 g L^–1^, enzymatic digest of soya bean 5 g L^–1^, sodium chloride 5 g L^–1^, glucose 2.5 g/L), and Luria–Bertani broth (tryptone 15 g L^–1^, yeast extract 5 g L^–1^, sodium chloride 10 g L^–1^) and placed in shaker incubators (150 r/min) at 4 and 15°C. Turbidity of the media after 2 weeks indicated bacterial growth. Different dilutions of the enrichment sample were plated on R2A, TSA, and LB agar plates using spread plate method. Plates were incubated at 4 and 15°C and observed daily for colony appearance. BGI-2 was among eight bacteria isolated from ice sample through sample enrichment. Strain was purified according to the streak plate method and cryopreserved at −80°C using 20% glycerol.

### Identification of Isolate

Preliminary identification of the isolate was done through colony morphology (size, elevation, margin, and pigmentation), cell morphology (Gram staining), and using different biochemical tests (triple sugar iron test, catalase, oxidase, citrate utilization, nitrate reduction). Whole genome sequencing of BGI-2 was done in BAS Lab, Institute of Marine and Environmental Technology, University of Maryland System Center for Environmental Sciences, using Illumina Miseq sequencing. Rapid Annotation using Subsystem Technology (Aziz et al., 2008) was used to get the complete 16S rRNA gene sequence and sequence was BLAST using EzBioCloud database (Yoon et al., 2017). 16S rRNA gene sequence was submitted to NCBI GenBank with the accession number MH681214. Phylogenetic tree was constructed by neighbor-joining method using MEGA 7.0 software (Saitou and Nei, 1987).

### Growth Optimization of BGI-2

To determine the optimal growth condition, BGI-2 was cultivated at different temperatures (4–45°C), pH (4–11), salinity (1–10%), carbon sources (glucose, galactose, lactose, sucrose, mannitol, mannose, maltose, arabinose, xylose, and starch), nitrogen sources (tryptone, peptone, yeast extract, urea, sodium nitrate, and ammonium sulfate), and glucose/yeast extract ratios (1/1, 10/1, 20/1, 30/1, 40/1, and 50/1). Effect of different concentration of molasses (1–5%) on the growth of BGI-2 was also investigated. Tryptic soya broth (17 g L^–1^ tryptone, 3 g L^–1^ soytone, 2.5 g L^–1^ glucose, 5 g L^–1^ sodium chloride, 2.5 g L^–1^ dipotassium hydrogen phosphate) was used as a growth medium for temperature and pH optimization. Tryptic soya broth amended with different NaCl concentrations was used for the salt requirement for optimal growth. For carbon utilization experiment, the mineral salt medium (MSM) was prepared according to Chayabutra and Ju (2000) (4 g L^–1^ NH_4_Cl, 2.5 g L^–1^ K_2_HPO_4_, 0.5 g L^–1^ NaCl, 0.3 g L^–1^ MgSO_4_×7H_2_O, 0.03 g L^–1^ FeCl_3_×6H_2_O, 0.01 g L^–1^ CaCl_2_, 0.01 g L^–1^ MnCl_2_×4H_2_O) supplemented with different sugars. All the sugars were filter sterilized using 0.22 μm syringe filters and aseptically added to the mineral salt media. All optimization experiments were carried out in 250 mL flasks with 50 mL working volume in a refrigerated shaker incubator (Innova 4340) at 150 r/min and 15°C. Optical density (OD_600_) was used to monitor the growth every 24 h up to 6 days. For optical density measurement, 48-well plates with 1 mL sample volume were used and readings were taken in a microplate reader (SpectraMax M5). All the tests were run in replicates. The growth rate was also determined by taking optical density (OD_600_) after every 2 h during the exponential phase of the growth.

### Optimization of EPS Production

The effect of environmental and nutritional conditions on EPS production by *Pseudomonas* sp. BGI-2 was investigated at different temperatures (4–45°C), pH (4–11), salinity (1–10%), incubation period (1–6 days), carbon sources (glucose, galactose, lactose, sucrose, xylose, arabinose, mannitol, mannose, maltose, glycerol, and starch), nitrogen sources (tryptone, peptone, yeast extract, urea, sodium nitrate, and ammonium sulfate), and glucose/yeast extract ratio (1/1, 10/1, 20/1, 30/1, 40/1, and 50/1). BGI-2 was also grown at different concentration (1–5%) of molasses to evaluate its effect on EPS production. All the experiments were conducted for 6 days at 15°C in a refrigerated shaker incubator (Innova 4340) at 150 r/min. Estimation of EPS was conducted daily using a microplate-based phenol sulfuric acid method (Le Parc et al., 2014).

### Extraction, Purification, and Estimation of EPS Production

**FIGURE 1:**
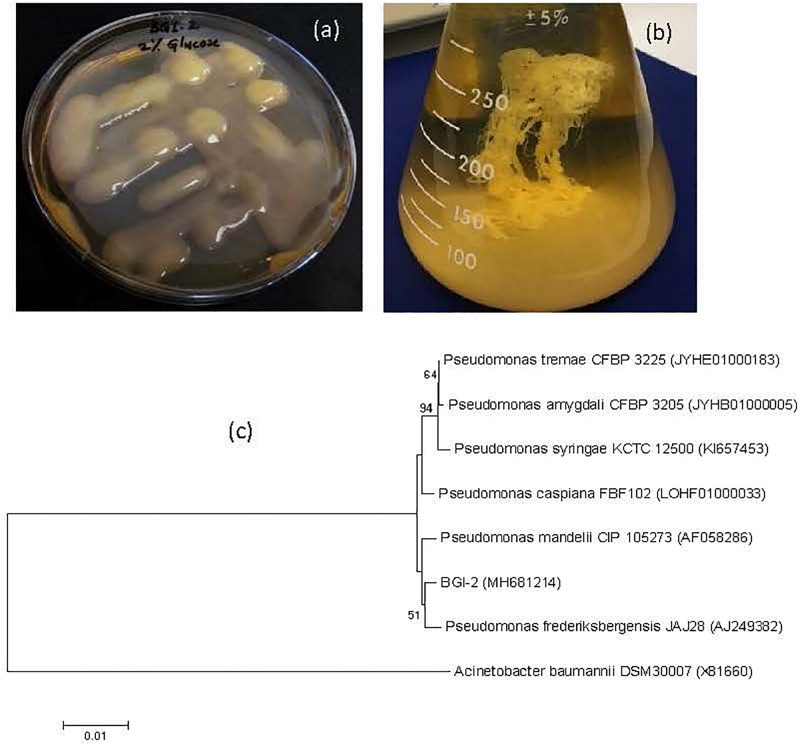
Culture and phylogeny **(a)** Mucoid phenotype of BGI-2 on tryptic soya agar supplemented with 2% glucose, **(b)** Ethanol precipitation of exopolysaccharide, and **(c)** phylogenetic tree of *Pseudomonas* sp. BGI-2 using neighbor joining method with bootstrap value (%) greater than 50 from 1000 replicates. Numbers in the brackets are GenBank accession numbers for the 16S rRNA gene sequences and *Acinetobacter baumannii* DSM 30007 is used as an outgroup to root the tree.

Extraction and purification of the EPS were conducted following the method used by Dabour and LaPointe (2005) with some modifications. For EPS extraction, bacterial culture samples were centrifuged (10,000 r/min, 4°C, 15 min) to pellet out the cells. EPS in the supernatant was precipitated using two volumes of chilled absolute ethanol and left overnight at 4°C (Figure 1b). The precipitated EPS was collected by centrifugation (10,000 r/min, 4°C, 15 min) and dried at room temperature. The crude EPS was dissolved in deionized water, treated with 12% (v/v) chilled trichloroacetic acid (TCA) and left at 4°C for 1 h. Precipitated proteins were removed by centrifugation (10,000 r/min, 4°C, 15 min). The deproteinized EPS in the supernatant was precipitated by addition of two volumes of chilled ethanol after overnight incubation at 4°C. The precipitated EPS was collected by centrifugation (10,000 r/min, 15 min, 4°C) and dried at room temperature. The EPS was re-dissolved in deionized water and dialyzed against distilled water for 2 days at 4°C using a dialysis membrane (Mw cut off: 12000 Da) to remove traces of TCA, salts, and low molecular weight molecules and freeze-dried in a lyophilizer (FreeZone 2.5, LABCONO). The purified EPS was re-dissolved in deionized water and EPS concentration was determined by phenol sulfuric acid method (Dubois et al., 1956). For rapid quantification, all the measurements were done in 96-well microplates using a microplate reader (Le Parc et al., 2014).

### Solubility Test

Solubility of the EPS was checked in different solvents including water, benzene, hexane, ethyl acetate, chloroform, methanol, ethanol, acetone, and DMSO.

### Analysis of Monosaccharide Composition of EPS Using High-Performance Anion-Exchange Chromatography With Pulsed Amperometric Detection (HPAEC-PAD)

Monosaccharide composition of the EPS was analyzed using high-performance anion-exchange chromatography with pulsed amperometric detection (HPAEC-PAD) adopted from the method used by Zhang et al. (2012). Hydrolysis of the EPS was carried out in trifluoroacetic acid (2N) at 100°C for 1 h (Maalej et al., 2015). Glucose, galactose, arabinose, mannose, glucosamine, galactosamine, glucuronic acid, *N*-acetylneuraminic acid (Neu5AC), and *N*-glycolylneuraminic acid (Neu5GC) were run as standard.

### NMR Spectroscopy of the EPS Preparations

800 MHz ^1^H NMR spectra with water suppression have been acquired from ∼1 mg EPS-BGI-2 in ∼400 mg CD_3_OD as described elsewhere (Hertkorn et al., 2013); further NMR experimental detail and acquisition parameters are shown in Supplementary Text T1.

### Freeze Thaw Survivability of EPS Producing *Pseudomonas* sp. BGI-2

*Pseudomonas* sp. BGI-2 was subjected to freeze thaw cycles to check its survivability/tolerance against freezing and freeze thaw cycles without the addition of any external cryoprotective agent. This test was performed by colony count method as described previously with some modifications (Sleight et al., 2006; Shivaji et al., 2013). Survivability of BGI-2 was compared with another psychrotrophic non-EPS producing *Rhodococcus* sp. BGI-11 isolated from the same environment (glacier ice) and a mesophilic *E. coli* k12. An overnight culture of these bacteria in late log phase was harvested through 5 min of centrifugation at 10,000 r/min. Cell pellets were re-suspended in 0.85% NaCl solution; 1 mL of each culture was transferred to sterilized cryovials. CFU/mL using spread plate method was done to determine the original cell numbers. All the vials were frozen at −80°C in a deep freezer. After 24 h, tubes were drawn out of the freezer and thawed for 25 min in a water bath at 25°C; 100 μL of thawed culture was serially diluted in 0.9% saline. Survivability of these bacteria was determined by comparing the log CFU counts before and after the freeze thaw cycles. BGI-2 and BGI-11 were plated on TSA plates whereas *E. coli* diluted culture samples were plated on LB plates. Tubes were again frozen at −80°C. This freeze thaw cycle was repeated after every 24 h for one week and CFU/ml for all the three bacteria were determined using the plate counting method. The test was run in replicates for all the three strains.

### Cryoprotective Activity of the EPS

The cryoprotective property of EPS using *E. coli* k12 as an indicator organism was determined by the method used by Dubey and Jeevaratnam (2015) with minor modifications. Precisely, an overnight culture of *E. coli* in late log phase was mixed with 1, 3, and 5% (w/v) EPS solution. The culture was first harvested through 5 min of centrifugation in a refrigerated centrifuge at 10,000 r/min. The cell pellets were re-suspended in 0.9% NaCl solution and used for testing. An equal volume of *E. coli* suspension in 1.5 mL cryo-vials was mixed with EPS solution to make a final volume of 1 mL. Now 100 μL samples from all the tubes were taken, serially diluted, and plated on LB plates using spread plate method to get the original bacterial count. All the vials were frozen at −80°C in a deep freezer. After 24 h, tubes were drawn out of the freezer and thawed for 25 min in a water bath at 25°C. The freeze thaw cycle was repeated after every 24 h for 1 week and CFU/mL of the indicator bacterium was determined using the plate count method. The survivability of *E. coli* k12 was determined by comparing the log CFU counts before and after the freeze thaw cycles. The relationship between the concentration of EPS and the frequency of freeze thaw cycles was also assessed. The cryoprotective effect of EPS was compared to 20% glycerol, which is a well-known cryoprotective agent for bacterial cryopreservation. *E. coli* culture without addition of a cryoprotective agent (EPS or glycerol) was used as negative control.

### Membrane Protection Assay

#### RBC Lysis Test

Membrane protection assay was performed by using human RBCs (erythrocytes) with a slight modified method as described previously (Sajjad et al., 2018b). RBCs were isolated by centrifugation of citrated blood at 2000 × *g* for 15 min. Cells were washed with saline phosphate buffer and then re-suspended in the same buffer up to the desired hematocrit level (1:10). RBCs were pretreated with two surface damaging agents including Triton-X and sodium dodecyl sulfate (SDS) as negative control. The % damage inhibition exhibited by the EPS was evaluated by adding 100 μL of EPS (5 mg/mL) to RBCs and incubation for 10 min, finally adding Triton-X and SDS to the reaction mixture. The final volume was adjusted to 200 μL and incubated for another 30 min. The reaction mixture was centrifuged at 5000 r/min for 5 min. The supernatant was collected and absorbance was determined at 540 nm. The percent hemolysis was calculated by the following formula:

%Hemolysis=(control-test/control)×100

#### Lipid Peroxidation Inhibition Assay

The thiobarbituric acid (TBA) assay was performed to assess malondialdehyde (MDA) concentration by the method described by Pérez et al. (2007) with some modification. Lipid peroxidation results in formation of MDA, a lipid peroxidation marker. The total lipid extract was recovered, according to a standard protocol as previously described (Bligh and Dyer, 1959); 250 μL of lipid samples from *E. coli* overnight grown culture was mixed with 125 μL of 20% TCA. The supernatant was collected and 100 μL of a 1% EPS solution was added and incubated for 10 min; 0.5 mL FeSO_4_ (0.07 M) was added to the mixture and incubated at 37°C for 1 h; 300 μL of this solution was mixed with 0.8% TBA reagent (200 μL), 8% SDS (200 μL) and incubated at 100°C for 1 h. The absorbance of chromophore was measured at 535 nm. The MDA concentration is presented as μM of MDA (standard curve *y* = 0.0947*x*−0.152) produced per mg of lipids using a molar extinction coefficient of 1.56 × 10^5^ M/cm (Konukoğlu et al., 1999; Sajjad et al., 2018a). The experiment was run in triplicate, and lipid extract without adding any EPS was used as a negative control.

## Results

### Screening and Identification of *Pseudomonas* sp. BGI-2

BGI-2 was isolated from the ice sample of Batura glacier, Pakistan. Direct plating of the melted ice sample on TSA, R2A, and LB agar plates did not yield any growth even after 9 months of incubation at 4°C. Isolation was done using sample enrichment in R2A broth before plating on R2A agar plates. The enrichment culture media turned turbid (indicating bacterial growth) after 2 weeks of incubation at 15°C. Plating of the enrichment sample on agar plates yielded eight morphologically different bacterial colonies after 1 month of incubation at 4 and 15°C. Colonies of BGI-2 appeared on Day 6 compared to other isolates which appeared only after 15–21 days. All the colonies were purified on separate plates and also cryopreserved at −80°C in 20% glycerol.

BGI-2 was the most abundant bacterial isolate, contributing more than 60% of all the colonies on each plate. BGI-2 produces large, circular, raised, and slimy colonies on agar plates. The unique feature of this glacial isolate was production of extremely mucoid colonies when grown on R2A or tryptic soy agar medium supplemented with 1–5% glucose (Figure 1a), indicating EPS production (Figure 1b). Gram staining revealed BGI-2 as a Gram negative bacterium with rod shape. BGI-2 demonstrated positive test for citrate utilization and negative for nitrate reduction. The triple sugar iron test revealed fermentation of glucose only and no fermentation was observed for sucrose and lactose. BGI-2 also demonstrated positive results for catalase and oxidase enzymes.

The complete sequence of 16S rRNA gene revealed strain BGI-2 clustered in to genus *Pseudomonas* (Figure 1c), with the nearest species: *Pseudomonas mandelii* (99.59%), *Pseudomonas frederiksbergensis* (99.59%), *Pseudomonas caspiana* (99.45%), *Pseudomonas tremae* (99.38%), and *Pseudomonas amygdali* (99.32%).

### Growth Optimization of *Pseudomonas* sp. BGI-2

BGI-2 was able to grow at a temperature range of 4–35°C. The optimal growth temperature for BGI-2 was suggested around 20–30°C. The highest growth rate was observed at 25°C with a mean exponential growth rate of 0.467 h^–1^ and generation time of 2.14 h. A decrease in growth rate with increase in generation time was observed at 4 and 35°C. No growth was observed at 45°C (Table 1). BGI-2 demonstrated good growth at a wide range of pH 5–11 (Table 1). The maximum growth was observed at pH 7 and pH 8. The mean growth rate at pH 7 was 0.397 h^–1^ (generation time 2.52 h) and at pH 8 was 0.399 h^–1^ (generation time 2.51 h). Decrease in growth rate toward the lower and higher pH was observed. No growth was observed at pH 4. Effect of different NaCl concentration on the growth revealed that BGI-2 is able to grow at a salt range of 1–5% (w/v). The maximum growth was observed in control (no NaCl) with a mean exponential growth rate of 0.39 h^–1^ (generation time 2.56 h) followed by 1% NaCl with a mean growth rate of 0.372 h^–1^ (generation time 2.69 h). The decrease in growth rate and increase in generation time was observed at the higher concentration of NaCl used. No growth was observed at 7% NaCl (Table 2). Effects of different concentrations (1–5%) of molasses (as a carbon source) on the growth revealed the maximum growth at 1% molasses, with a mean growth rate of 0.318 h^–1^ (generation time 3.14 h) followed by 3 and 5% molasses. The least growth was observed in the control with no addition of molasses. BGI-2 strain was able to utilize glucose, galactose, mannose, mannitol, glycerol, and molasses as carbon source. No growth was observed when lactose, sucrose, xylose, arabinose, and maltose were used as carbon source. For nitrogen source, the maximum growth was observed for yeast extract followed by peptone and tryptone (Table 3). Poor growth was recorded for inorganic nitrogen sources such as sodium nitrate and ammonium sulfate. For glucose/yeast extract ratios, the maximum growth was observed at 10/1 followed by 20/1 and 1/1. Growth decreased at the higher glucose/yeast extract ratio (Table 3).

**TABLE 1:** Effect of different temperatures (4–45°C) and pH (5–11) on the growth and EPS production by *Pseusomonas* sp. BG1-2.

**Temperature**			**pH**		
**Temperature (°C)**	**OD_600_**	**EPS (mg L^–1^)**	**pH**	**OD_600_**	**EPS (mg L^–1^)**
4	6.74 ± 0.14	279 ± 22	5	6.95 ± 0.01	210 ± 9
15	7.69 ± 0.10	283 ± 7	6	7.04 ± 0.06	273 ± 43
25	8.81 ± 0.23	209 ± 12	7	6.80 ± 0.11	263 ± 31
35	4.08 ± 0.18	−	8	7.01 ± 0.09	256 ± 23
45	–	−	9	6.64 ± 0.06	228 ± 56
			10	6.06 ± 0.01	144 ± 25
			11	4.83 ± 0.02	−

*The data are presented as the mean value ± standard deviation of duplicate readings (– means no growth or EPS production).*

**TABLE 2:** Effect of different salinities (1–7%) and molasses (1–5%) on the growth and EPS production by *Pseusomonas* sp. BG1-2.

**Salinity**			**Molasses**		
**NaCl (%)**	**OD_600_**	**EPS (mg L^–1^)**	**Molasses (%)**	**OD_600_**	**EPS (mg L^–1^)**
Control	7.28 ± 0.04	198 ± 9	Control	6.83 ± 0.35	130 ± 10
1	7.90 ± 0.18	287 ± 4	1	7.50 ± 0.42	296 ± 23
3	6.61 ± 0.07	227 ± 8	3	7.82 ± 0.22	451 ± 10
5	3.25 ± 0.15	161 ± 18	5	8.50 ± 0.54	675 ± 59
7	–	−			

*The data are presented as the mean value ± standard deviation of duplicate readings (– means no growth or EPS production).*

**TABLE 3:** Effect of different nitorgen sources and glucose/yeast extract on the growth and EPS production by *Pseusomonas* sp. BG1-2.

**Nitrogen sources**			**Glucose/yeast extract**		
	
**N compounds**	**OD_600_**	**EPS (mg L^–1^)**	**Glucose/yeast extract**	**OD_600_**	**EPS (mg L^–1^)**
Tryptone	5.77 ± 1.04	217 ± 20	1/1	6.41 ± 0.18	234 ± 21
Peptone	7.40 ± 0.08	228 ± 26	10/1	11.58 ± 0.07	612 ± 19
Yeast Extract	9.45 ± 0.22	375 ± 23	20/1	8.70 ± 0.14	511 ± 14
Urea	0.498 ± 0.06	–	30/1	5.27 ± 0.08	355 ± 15
(NH_4_)_2_SO_4_	1.80 ± 0.19	–	40/1	3.94 ± 0.19	152 ± 8
NaNO_3_	0.791 ± 0.01	–	50/1	2.85 ± 0.04	−

*The data are presented as the mean value ± standard deviation of duplicate readings (– means no growth or EPS production).*

### EPS Production by *Pseudomonas* sp. BGI-2

The maximum production of EPS was recorded at 15°C (283 mg L^–1^) during 96 h. Likewise growth, EPS production was low at 4°C during the first 72 h and reached to the maximum during 144 h (279 mg L^–1^). The maximum production of EPS at 25°C was recorded around 209 mg L^–1^. No EPS production was observed at 35°C and above (Table 1). The pH seems to have the least effect on the production of EPS. The maximum EPS production was observed at pH 6 (273 mg L^–1^), followed by pH 7 (263 mg L^–1^) and pH 8 (256 mg L^–1^) during the early stationary phase of growth. No EPS production was observed at the extremes of pH (pH 4 and pH 11) (Table 1). For salinity, the maximum EPS production was observed at 1% NaCl (287 mg L^–1^) followed by 3% NaCl (227 mg L^–1^). Likewise growth, no EPS production was observed for the treatment with 7% NaCl (Table 2). Effect of different concentrations of molasses on EPS production revealed the maximum EPS produced at 5% molasses (675 mg L^–1^) followed by 3% (451 mg L^–1^) and 1% (296 mg L^–1^). Least EPS production was observed in the control (Table 2). Although the strain BGI-2 demonstrated growth in MSM media supplemented with glucose, galactose, mannose, mannitol, glycerol, and molasses; EPS production was negligible in the minimal medium. For nitrogen source, the maximum EPS production was recorded for yeast extract (375 mg L^–1^) followed by peptone (228 mg L^–1^) and tryptone (217 mg L^–1^) (Table 3). Likewise the growth, no EPS production was recorded for the inorganic nitrogen sources (sodium nitrate and ammonium sulfate). For glucose/yeast extract ratio, the maximum EPS production was recorded at 10/1 (612 mg L^–1^) followed by 20/1 (511 mg L^–1^) and 30/1 (355 mg L^–1^) (Table 3).

Putting all the optimized nutritional and environmental conditions in one experiment resulted in a high yield of EPS 2.01 g L^–1^ (Table 4). The optimized conditions included temperature (15°C), pH (6), NaCl (10 g L^–1^), glucose as carbon source (100 g L^–1^), yeast extract as nitrogen source (10 g L^–1^), and glucose/yeast extract (10/1).

**TABLE 4:** EPS production by BGI-2 under optimized conditions (15°C, pH 6, 10 g L^–1^ NaCl, 100 g L^–1^ glucose, 10 g L^–1^ yeast extract, and glucose/yeast extract ratio 10/1).

**Incubation time (hours)**	**OD_600_**	**EPS (mg L^–1^)**
24	3.63 ± 0.16	1510 ± 13
48	5.44 ± 0.04	1694 ± 31
72	7.19 ± 0.10	1759 ± 33
96	8.16 ± 0.11	2010 ± 35
120	8.74 ± 0.24	1654 ± 47
144	9.08 ± 0.28	1450 ± 31

The effect of incubation period on the growth as well as EPS production was also studied. For most of the optimization experiments, the maximum growth was observed from 24 to 48 h and EPS production from 72 to 96 h. Both the growth and EPS yield declined afterward.

### Solubility in Different Solvents

The EPS was completely soluble in water; partially soluble in benzene, n-hexane, ethyl acetate, chloroform; insoluble in methanol, ethanol, acetone, and DMSO.

### Monosaccharide Composition of EPS Using High-Performance Anion Exchange Chromatography With Pulsed Amperometric Detection (HPAEC-PAD)

**FIGURE 2:**
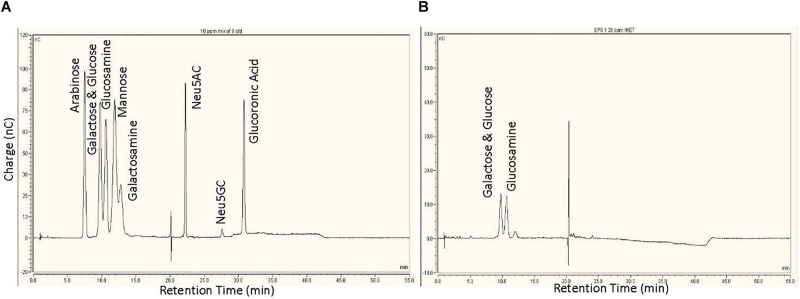
Monosaccharide analysis of the exopolysaccharide: **(A)** High-performance anion exchange chromatography with pulsed amperometric detection (HPAEC-PAD) chromatogram of nine sugars used as standard. **(B)** HPAEC-PAD chromatogram of monosaccharides present in EPS.

Analysis of the monosaccharide composition is very critical in characterizing the structure of complex carbohydrates. The sugar monomers were characterized by appearance of peaks at different retention time when compared to the standards (Figure 2A). Results revealed the EPS is composed mainly of three sugar monomers, including glucose, galactose, and glucosamine (Figure 2B).

### NMR Spectrometry of EPS

**FIGURE 3:**
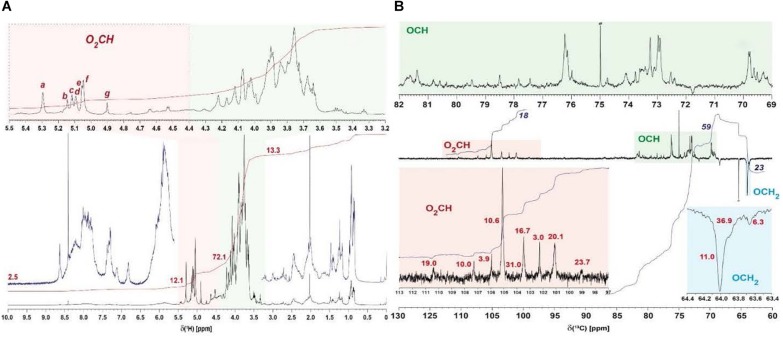
NMR analysis of the exopolysaccharide. **(A)** ^1^H NMR spectra of EPS preparation BGI-2 (800 MHz, D_2_O), with expansion of carbohydrate-derived NMR resonances shaded in orange (anomeric O_2_CH-units) and green (OCH and OCH_2_ units); major anomeric ^1^H NMR resonances a–g are annotated (cf. text); numbers denote relative ^1^H NMR section integrals (total: 100%), and **(B)** ^13^C DEPT NMR spectra of EPS preparation BGI-2 (125 MHz, D_2_O), with expansion of carbohydrate-derived ^13^C NMR resonances shaded in orange (anomeric O_2_CH-units) and green (OCH-units) and blue (OCH_2_-units) units; red numbers indicate half-width (Hz) of anomeric and OCH_2_^13^C NMR resonances, most probably resulting from overlap of related but different chemical environments; blue numbers denote relative ^13^C NMR section integrals (total: 100%, with respect to section shown).

^1^H NMR spectra acquired from several EPS samples indicated the presence of very similar carbohydrates in all EPS preparations, with variable proportions of attendant aromatic and aliphatic compounds (data not shown). In all preparations of EPS, all carbohydrate-related NMR resonances showed only rather minor variations of relative NMR amplitude (<10%) at virtually identical δ_H_ (data not shown). This referred to the relative proportions of anomeric protons as well (Table 5) and implied production of an EPS molecule or a mixture of EPS molecules of rather uniform composition and structure, irrespective of details of culture conditions. The isolate BGI-2 showed seven major ^1^H NMR resonances (denoted a–g in Figure 3A) of anomeric O_2_CH-units, the ^1^H NMR integral ratio of (OCH + OCH_2_)/O_2_CH units was ∼6, suggesting the presence of peptide-derived CONH-CαH NMR resonances as well (Supplementary Figure S1).

**TABLE 5:** Relative areas of seven major NMR resonances (800 MHz, D_2_O) of anomeric positions (O_2_CH-units) in BGI-2 computed from lineshape-fitting of 1D ^1^H NMR spectra.

**Number of anomeric HSQC cross peak**	**δ (^1^H) [ppm]**	**width [Hz]**	**% area**	**δ (^13^C) [ppm]**	**Nominal count of anomeric positions**
A	5.301	9.4	19.0	103.45	4
B	5.151	7.7	8.3	105.14	1
C	5.123	9.2	14.8	101.14	3
D	5.098	10.0	15.3	101.12	3
E	5.062	5.9	13.1	105.08	2
F	5.052	7.9	22.7	105.08	5
G	4.907	5.5	6.6	102.32	1

*The nominal count refers to proposed minimum numbers of defined anomeric CH-units in a complex EPS, when this EPS would be a single compound. δ_*C*_ is derived from ^1^H, ^13^C HSQC NMR spectra.*

^1^H, ^1^H TOCSY (total correlation spectroscopy) and ^1^H, ^13^C HSQC NMR spectra (heteronuclear single quantum coherence) showed that the major cross peaks of the EPS preparations divided into those derived from carbohydrates and those arising from peptides (Supplementary Figure S1). The carbohydrates in EPS were itself complex, with >15 HSQC resolved cross peaks representing anomeric O_2_CH-groups in ^1^H, ^13^C HSQC NMR spectra (Supplementary Figure S1B). Among those, seven major cross peaks (peaks a–g; Supplementary Figure S1B) suggest either the presence of a mixture of several carbohydrates or the presence of a single, uniform EPS molecule. If that would apply, a minimum of 19 anomeric positions is expected for this EPS (Table 5). It is, however, more likely that our current EPS isolates represent mixtures of different carbohydrate molecules at given ratios of abundance; an alternative option is the presence of some carbohydrate oligomers with repetitive connectivities but different overall size.

^13^C DEPT-135 NMR spectra (distortionless enhancement by polarization transfer) showed only protonated carbon atoms at a larger S/N ratio than available from single-pulse ^13^C NMR spectra and allowed distinction of methylene (negative amplitude) and methine carbon atoms (positive amplitude) in carbohydrates. They indicated the presence of four major (groups of) carbohydrates as deduced from their distinct anomeric (O_2_CH) ^13^C NMR resonances, and several minor ones. The variable linewidth of the NMR resonances produced by anomeric carbon atoms most likely resulted from superposition of similar chemical environments in complex EPS (Figure 3 and Supplementary Figure S2C) rather than from intrinsic effects of local mobility of defined chemical environments in pure polysaccharides (here, less mobile fragments would have produced NMR resonances with larger linewidths). The superposition of distinct anomeric positions in resonances of ^13^C NMR spectra was also corroborated by ^1^H, ^13^C HSQC NMR cross peaks (Supplementary Figure S1B); the seven major HSQC NMR cross peaks a–g projected on the four major ^13^C NMR resonances (Figure 3B and Supplementary Figure S2C).

### Survivability of *Pseudomonas* sp. BGI-2 After Freeze Thawing Without Addition of an External Cryoprotective Agent

**FIGURE 4:**
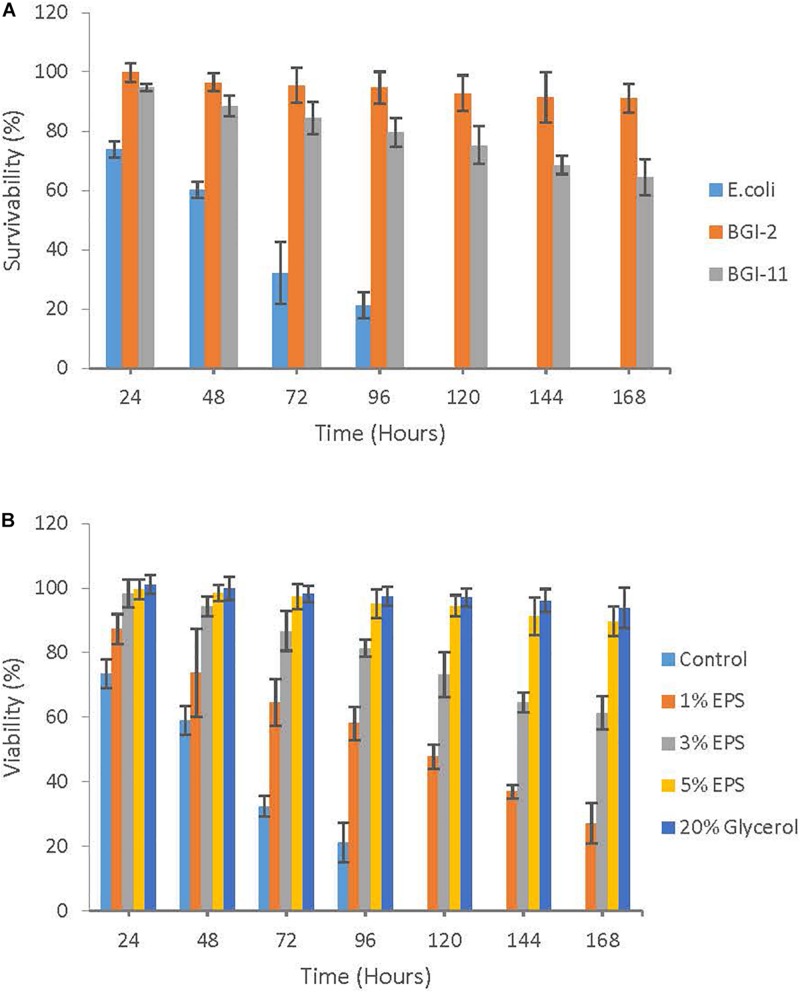
Role of EPS in cryoprotection: **(A)** Freeze thaw survivability of *Pseudomonas* sp. BGI-2 compared to *Rhodococcus* sp. BGI-11 and *E. coli* k12 using plate count method. **(B)** Cryoprotective effect of different concentrations of EPS (1–5%) on *E. coli* k12 subjected to seven freeze thaw cycles using plate count method.

The freeze thaw survivability of BGI-2 was compared to another non-EPS producing psychrotrophic strain *Rhodococcus* sp. BGI-11 isolated from the same environment and a mesophilic *E. coli* k12 strain. After seven freeze thaw cycles, survivability of BGI-2 was 91.1% compared to 64.5% in BGI-11. Survivability of *E. coli* decreased significantly with the increase in freeze thaw cycles and no viability was observed after the fourth cycle. Overall survivability decreased with an increase in the freeze thaw events to variable degree depending on the strains (Figure 4A). However, BGI-2 demonstrates the maximum tolerance compared to the other two bacteria when subjected to repeated freeze thaw cycles.

### Cryoprotective Activity of EPS

*Escherichia coli* k12 was used as an indicator organism for the cryoprotection assay. We found that *E. coli* k12 strain recovered to varying degrees which depended on the concentration of EPS used and the number of freeze thaw cycles the strain was subjected. Survivability of *E. coli* k12 increased significantly with an increase in EPS concentration from 1 to 5% (w/v). The maximum survivability after seven freeze thaw cycle was observed for 5% EPS (89.7%) followed by 3% EPS (61.5%) and 1% EPS (27.2%). Survivability was severely affected in the control (no EPS) with no viability observed after 4 freeze thaw cycles. Glycerol had the highest recovery with a survivability rate of 94.0% (Figure 4B).

### Membrane Protection Assay (RBCs Lysis)

**FIGURE 5:**
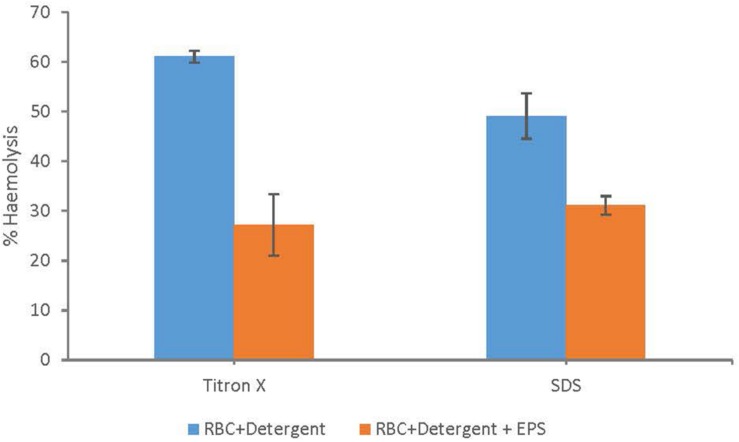
Membrane protection assay (RBCs lysis) using human RBCs shows significant reduction in% hemolysis when RBCs were pretreated with EPS compared to the controls with no EPS but only the surfactants (sodium dodecyl sulfate and titron x).

Membrane protection assay using RBCs lysis revealed that addition of EPS resulted in a significant reduction in % hemolysis (Figure 5) compared to the controls with only the lysing agents (Triton-X and SDS) and no addition of EPS. These detergents are surface damaging substances which resulted in the lysis of RBCs membrane up to 61% (Triton-X) and 49% (SDS), respectively. However, pre incubation of RBCs with EPS resulted in a greater protection of the RBCs membrane. Overall, addition of EPS resulted in 50% and 34% reduction in hemolysis in the presence of Triton-X and SDS as the lysing agents (Figure 5).

### Lipid Peroxidation Assay

FeSO_4_ and other reactive substances-induced oxidative stress causes damage to cellular lipids and proteins that ultimately results in cell death. The effect of EPS on cellular lipids peroxidation inhibition was measured. Our results showed that lipid content of *E. coli* without EPS displayed significant damage to its cellular lipids. Lipid peroxidation was noticed up to 1.96 μM/mg in negative control which was reduced to 1.27 μM/mg using EPS. A clear difference in lipid peroxidation inhibition with EPS and without EPS was observed, revealing the protective role of EPS as cell protectant.

## Discussion

*Pseudomonas* sp. BGI-2 was selected for further studies based on its high abundance, relatively rapid growth at low temperatures, and maximum EPS production compared to the other glacial isolates. When glacier ice enrichment samples were streaked on agar plates, BGI-2 made up more than 60% of all bacterial colonies. Colonies of BGI-2 appeared on agar plates within the first week of plating enrichment samples whereas it took 2–3 weeks for the other bacterial isolates to form visible colonies. The initial observations suggested how well this strain is adopted to the harsh environment characterized by frequent freeze thaw cycles, desiccation (low water activities), high UV radiation, and low nutrient availability. Molecular characterization using complete 16S rRNA gene sequencing placed BGI-2 in the genus *Pseudomonas* with the closest species *P. mandelii* and *P. frederiksbergensis*. Recently, Kumar et al. (2019) isolated psychrotrophic strain *P. frederiksbergensis* ERDD5:01 from a glacial stream in the Himalaya and demonstrated its high survivability/tolerance to stress conditions such as freezing, freeze thaw cycles, and UV-C radiations. *Pseudomonas* species are found in diverse environment due to their great metabolic flexibility. Cold-adapted *Pseudomonas* species have been isolated from different cold environments around the world including the polar and non-polar regions (Jang et al., 2012; Sathiyanarayanan et al., 2016; Vasquez-Ponce et al., 2017). Männistö and Häggblom (2006) characterized the psychrotophic heterotrophic bacteria from Finish Lapland and found one third of the isolates as *Pseudomonads*, representing 60% of all isolates from alpine tundra soils. Meyer et al. (2004) found high prevalence of psychrotrophic *Pseudomonas* in alpine soil samples constituting key members of the bacterial community.

Temperature is an important factor determining the growth and metabolite productions in any organism. *Pseudomonas* sp. BGI-2 grew well at a temperature range of 4–35°C. Since the growth rate was low at 15°C compared to 25°C and decreased above 30°C, we assume therefore the optimum growth range lies within 20–30°C. Morita (1975) defined psychrophiles as organisms having an optimal growth temperature around 15°C or below and maximal growth temperature of 20°C. Psychrotrophs were defined as organisms able to grow at low temperatures but have their optimal growth temperature above 20°C. According to these definitions BGI-2 falls in the category of psychrotrophs which is obvious from the fact that it was isolated from an environment that undergoes frequent temperature changes.

Although the optimum temperature for BGI-2 lies between 20 and 30°C, the maximum EPS production was observed at suboptimal temperatures 15 and 4°C. Nichols et al. (2005) found a similar result while investigating the effect of incubation temperature on the growth and EPS production of a psychrotolerant sea ice bacterium. Their study revealed that EPS yields at −2 and 10°C were 30 times higher than at 20°C, which is the optimum growth temperature for many psychrotolerant strains. Most of the optimization experiments in our study revealed that the maximum production of EPS took place in the stationary phase, which is in accordance with the previous studies (Samain et al., 1997; Moriello et al., 2003; Nichols et al., 2005).

Carbohydrates and individual sugars are the preferred carbon sources for EPS production. Among the many substrates tested, BGI-2 was able to use a broad range of carbon sources including glucose, galactose, mannose, mannitol, glycerol, and molasses. The metabolic versatility of *Pseudomonas* facilitates its survival in different habitats and adaptation to varied environmental conditions. Although BGI-2 was able to grow in MSM supplemented with glucose or galactose as the carbon source, growth and biomass production was not as good as in a nutrient rich medium (tryptic soy broth). Growth and biomass production of BGI-2 was two to three times higher in tryptic soy broth than in MSM. Also, EPS production in MSM supplemented with either 1% glucose, galactose, or molasses was negligible compared to the nutrient rich medium (modified tryptic soy both) where EPS yield was as high as 2.01 g L^–1^. Tryptic soy broth has other organic nutrients to support the bacterial growth and biomass production. Production of metabolic products is very much dependent on the nutritional requirement of the bacteria and EPS production is highly influenced by the carbon and nitrogen sources in the fermentation medium. In our study, organic nitrogen sources (yeast extract, peptone, and tryptone) outperformed the inorganic nitrogen sources (sodium nitrate and ammonium sulfate) in terms of cell growth and EPS production. These results are in accordance with a previous study, in which the growth and EPS yield from the *Pseudomonas* species were significantly higher in the presence of the organic nitrogen compounds compared to the inorganic nitrogen sources (Maalej et al., 2015). According to Bajaj et al. (2006), organic nitrogen sources are complex and contain amino acids and vitamins which improve the cell growth and EPS yield. Therefore, production of EPS is influenced greatly by the carbon and nitrogen sources used.

Under optimized conditions, EPS production by *Pseudomonas* sp., BGI-2 was 2.01 g L^–1^ which is significantly higher for a *Pseudomonas* species previously reported using the same method for EPS quantification (phenol – sulfuric acid method). Celik et al. (2008) optimized EPS production for two *Pseudomonas* species and found xylose as the preferable carbon source. The maximum EPS yield for *Pseudomonas aeruginosa* G1 was recorded at 3% xylose (368 mg L^–1^) and *Pseudomonas putida* G12 at 2% xylose (262 mg L^–1^). In another study, the maximum production of EPS under optimized conditions for a chromium-resistant *P. aeruginosa* was recorded as 863 mg L^–1^ (Kılıç and Dönmez, 2008).

The choice of a carbon source in culture medium is critical and can be sugar or non-sugar depending on the strain. Sugar waste molasses can be used as a cheap carbon source for EPS production. The strain BGI-2 grew rapidly and produced high EPS yield in the medium supplemented with molasses as carbon source. The production cost is still a major obstacle for large scale production of many promising EPSs. The major nutrient for EPS production is the carbon sources used (mainly sugars) making the process costly. It is therefore important to optimize fermentation conditions and substitute nutrients with less expensive carbon substrates. Molasses, a waste product from the sugar refinery industry, is not only rich in sugars but also contains other essentials minerals which can be helpful in bacterial growth and EPS production. Using molasses as a cheap carbon substrate for EPS production has been reported previously (Küçükaşik et al., 2011). Strain BGI-2 also was able to use glycerol as a carbon source. Crude glycerol is generated as a waste in the biodiesel industry and can be employed as a cheap source of carbon for large-scale EPS production.

EPS from extremophiles is considered as a stress molecule which is thought to assist microorganisms to cope with the extremes of temperatures, salinity, and desiccation (Tamaru et al., 2005; Bemal and Anil, 2017; Casillo et al., 2017). Therefore, the survivability of BGI-2 against a series of freeze thaw cycles was compared to two other bacteria., including a non-EPS producing bacterium *Rhodococcus* sp., BGI-11 isolated from the same environment and a mesophilic *E. coli* k12. Survivability of EPS producing BGI-2 was significantly higher than that of BGI-11 and *E. coli*. High survivability of the EPS producing BGI-2 compared to the non-EPS strains suggests a role of EPS in protecting cells from damages caused by the freezing conditions and freeze thaw events. Krembs et al. (2002) found high concentration of exopolymeric substances in brine channels of the Arctic sea ice. They suggested that the exopolymeric substances could have been produced by the active bacteria and diatoms at this very low temperature and high salinity environment for cryoprotection. Casillo et al. (2017) demonstrated the cryoprotective role of an EPS from cold-adapted bacterium *Colwellia psychrerythraea* 34H. Tamaru et al. (2005) studied the role of extracellular polysaccharides in desiccation and freeze tolerance in the terrestrial cyanobacterium *Nostoc commune.* They demonstrated that the EPS depleted cells were sensitive to desiccation and freeze thaw treatment compared to when the EPS was intact. Similarly, Aslam et al. (2012) proposed EPS production as a strategy by the polar diatoms for survival in the cold and saline environment of the sea ice.

The effect of EPS on survivability of *E. coli* k12 after a series of freeze thaw cycles implied its role in cryoprotection. Survivability of *E. coli* depended on the concentration of EPS used and the number of freeze thaw cycles to which the strain was subjected. Survivability of *E. coli* k12 strain was improved as the concentration of EPS increased from 1 to 5% (w/v). Survivability was severely affected in the treatment with no addition of EPS where no viability was observed after four freeze thaw cycles. This is obvious as a freeze thaw cycle is injurious to cells, and bacteria inhabiting cold environments which undergo temperature fluctuation must adopt some strategies to cope with the change in temperature. BGI-2 was isolated from an environment that experiences large temperature fluctuations with freezing nights and temperatures that can go up to 10°C in the day time resulting in repeated freeze thaw events. EPSs are thought to work as a cryoprotectant in such situations. The EPS demonstrated high cryoprotective activity based on the survivability of a mesophilic *E. coli* strain. Survivability in 5% EPS was comparable to 20% glycerol which is a common cryoprotectant used for bacterial cryopreservation. The addition of EPS significantly improved the tolerance of *E. coli* to repeated freeze thaw cycles, indicating an universal cryoprotective role. These results are in accordance with previous studies which also demonstrated the cryoprotective role of bacterial EPSs (Marx et al., 2009; Liu et al., 2013; Carrion et al., 2015; Dubey and Jeevaratnam, 2015). In addition, we did further test to link the role of EPS to membrane stability. The RBCs lysis assay revealed significant reduction (∼50%) in hemolysis caused by the surfactants, when cells were pre-treated with the EPS. This was further validated by the lipid peroxidation inhibition assay where significant decrease in lipid peroxidation was observed when the *E. coli* lipid was treated with EPS. The lipid content of *E. coli* without EPS displayed significant damage to its cellular lipids, revealing the role of EPS as the cellular lipid protectant. Reactive substances result in oxidative damage to the cellular lipids leading to cell death. To the best of our knowledge, this is the first study where a microbial EPS was used for an improved reduction in hemolysis against membrane surface lysing agents (detergents). However, the inhibition of lipid peroxidation using EPS from microbial origin has been reported previously (Guo et al., 2010; Chen et al., 2011). The inhibitory effect of EPS from psychrophiles on lipid oxidation is very critical as oxidative stress increases at low temperature (Chattopadhyay et al., 2011). This could be one reason for BGI-2 to successfully colonize the glacier ice.

The current NMR data and auxiliary information are not sufficient for a definite analysis of EPS structural detail. This in part resulted from the paucity of meaningful cross peaks in ^1^H, ^13^C HMBC NMR spectra of BGI-2 (heteronuclear multiple bond correlation; data not shown) which precluded definition of inter-residue linkages. Weak HMBC cross peaks might in part have resulted from fast transverse NMR relaxation which could indicate the presence of rather larger size EPS molecules. ^1^H, ^13^C HSQC-TOCSY (data not shown) and standard 2D ^1^H, ^1^H TOCSY and 2D ^1^H, ^1^H NOESY (Supplementary Figures S2, S3) and selective 1D ^1^H NMR experiments (nuclear overhauser effect spectroscopy) were inconclusive as well (Supplementary Figure S4). However, absence of major acetyl groups (δ_H_ ∼ 2 ppm, would appear as singlet resonance) and other relevant lipid NMR resonances (Turska-Szewczuk et al., 2014) is proposed from NMR integral ratios of anomeric and the respective aliphatic NMR resonances; HSQC cross peaks are in agreement with galactose and glucose derivatives (Landersjö et al., 2002; Seviour et al., 2010; Gerwig et al., 2013). Rhamnose will not be part of a major EPS molecule, because the conspicuous methyl NMR resonance (δ_H_ ∼ 1.3 ppm; Casillo et al., 2015) is virtually absent in all EPS samples.

## Conclusion

*Pseudomonas* sp. BGI-2 is a promising strain able to grow at a wide range of temperatures and pH. BGI-2 can be used as a producer strain for the production of a cryoprotective membrane stabilizing EPS. The strain produces high yields of a cryoprotective EPS at low temperatures and can use molasses or glycerol as cheap sources of carbon for large-scale production. Cryopreservation of cells and tissues is an essential tool in biotechnology and medicine, and further research can pave the way for EPS-BGI-2 as an effective cryoprotective agent. Cold-adapted microorganisms offer many beenefits over their mesophilic counterparts through energy saving such as production of essential metabolites at low temperature, negating the requirement for expensive heating steps, rapid and easy inactivation of their enzymes as they are heat-labile. Working at low temperature also minimizes undesirable chemical reactions and contaminations that usually occur at higher temperatures. Microbial EPSs, being natural polymers, are considered ecofriendly due to its biodegradability and non-toxicity, compared to the recalcitrant chemical cryoprotective compounds currently in use.

## Data Availability Statement

The datasets generated for this study can be found in 16S rRNA accession number MH681214.

## Author Contributions

NH and MG did the NMR analysis of the EPS sample including data interpretation. WS did the HPLC analysis of the EPS sample including data analysis. AS and FH were involved in the design of the study including sampling of ice sample to bacterial isolation. FC supervised throughout this project and helped in the writing of the manuscript. PA did the experimentation work, data analysis, and manuscript write up.

## Conflict of Interest

The authors declare that the research was conducted in the absence of any commercial or financial relationships that could be construed as a potential conflict of interest.
