# Supplementary material for: A Glacier Bacterium Produces High Yield of Cryoprotective Exopolysaccharide
Source: Front Microbiol. 2020 Feb 11;10:3096. doi: 10.3389/fmicb.2019.03096 (PMC7026135; doi:10.3389/fmicb.2019.03096)
Supplement: Supplementary file 1 [file Data_Sheet_1.pdf]

A Glacier Bacterium Produces High Yield of Cryoprotective Exopolysaccharide

Pervaiz Ali[1,2], Aamer Ali Shah[2], Fariha Hassan[2], Norbert Hertkorn[3], Michael Gonsior[4], Wasim Sajjad[5], and Chen Feng[1*]

[1] Institute of Marine and Environmental Technology, University of Maryland Center for Environmental Science, Baltimore, MD, United States, [2] Applied Environmental and Geomicrobiology Laboratory, Department of Microbiology, Quaid-i-Azam University, Islamabad, Pakistan, [3] Research Unit Analytical Biogeochemistry, Helmholtz Zentrum München, Munich, Germany, [4] Chesapeake Biological Laboratory, University of Maryland Center for Environmental Science, Baltimore, MD, United States, [5] Department of Biological Sciences, National University of Medical Sciences, Rawalpindi, Pakistan

#Corresponding Author: chenf@umces.edu, +1-410-234-8866, FAX number: +1-410-234-8896

Supplementary Material

NMR spectroscopy of EPS preparations (Material and Methods)

1-5 mg of solid EPS preparation were suspended in ~ 600 µL D_2O (100% ^{2}H; Merck, Darmstadt) with minuscule amounts of deuterated TMSP ($(H_3C)_3Si-CD_2-CD_2-COONa$, δ_H = 0 ppm) added as reference and subjected to 5 minutes of ultrasonic bath. Not all EPS was dissolved under these conditions, but NMR spectra of EPS solution looked rather alike when prepared from different ratios of solid EPS and D_2O. The supernatant was collected after centrifugation and the solution was transferred to Bruker match tubes ranging from 2.5-5 mm diameter, and sealed; typical solubility was estimated to ~30%. NMR spectra of aqueous EPS extracts were acquired with a Bruker Avance III NMR spectrometer at 800.13 MHz (B_0 = 18.7 T) at 300 K with a 5 mm z-gradient ^{1}H / ^{13}C / ^{15}N / ^{31}P QCI cryogenic probe (90° excitation pulses: ^{13}C ~ ^{1}H ~ 10 µs). 1D ^{1}H NMR spectra were recorded with a spin-echo sequence (10 µs delay) to allow for high-Q probe ringdown, and classical pre-saturation to attenuate the residual water present "*noesypr1d*", typically 512 scans (5 s acquisition time, 5 s relaxation delay, 1 ms mixing time; 1 Hz exponential line broadening). Lorentzian lineshape fitting was performed with Bruker TopSpin software 2.3pl7. Selective excitation was performed with Bruker standard pulse sequences *"selnogpzs"* and *"seldigpzs"*, with aq = 3 s, d1 = 0.1s, TOCSY mixing time d9 = 250 ms, NOESY mixing time d8 = 350 msec and 400 scans each. The one-bond coupling constant ^{1}J(CH) used in the 2D ^{1}H,^{13}C DEPT-HSQC NMR spectrum (*hsqcedetgpsisp2.2*) was set to 145 Hz; other conditions: ^{13}C 90 deg decoupling pulse, GARP (70 µs); 50 kHz WURST 180 degree ^{13}C inversion pulse (Wideband, Uniform, Rate, and Smooth Truncation; 1.2 ms); F2 (^{1}H): spectral width of 5981 Hz (11.96 ppm); 1.25 s relaxation delay; F1 (^{13}C): SW = 17607 Hz (140 ppm). HSQC-derived NMR spectra were computed to a 8192 × 1024 matrix. Phase sensitive echo-antiecho TOCSY spectra (*dipsi2etgpsi*) and phase sensitive NOESY spectra (*noesygpphpp*; aq = 1 s, d1 = 1.5 s, mixing time = 350 ms) used a spectral width of 5498 Hz and were computed to a 16384 × 2048 matrix. The ^{13}C DEPT NMR spectrum was acquired with a Bruker Avance III NMR spectrometer at 125.77 MHz (B_0 = 11.7 T) at 300 K with a 5 mm z-gradient dual ^{13}C / ^{1}H / dual cryogenic probe (90° excitation pulses: ^{13}C ~ ^{1}H ~ 12 µs), with an acquisition time of 1 s and a interpulse delay of 2 s.

Reference for Fig. S1:

N. Hertkorn, A. Permin, I. Perminova, D. Kovalevskii, M. Petrosyan, A. Kettrup,
Comparative Analysis of Partial Structures of a Peat Humic and Fulvic Acid Using One- and Two Dimensional Nuclear Magnetic Resonance Spectroscopy,
J. Environ. Qual. **31** (2002) 375-387.

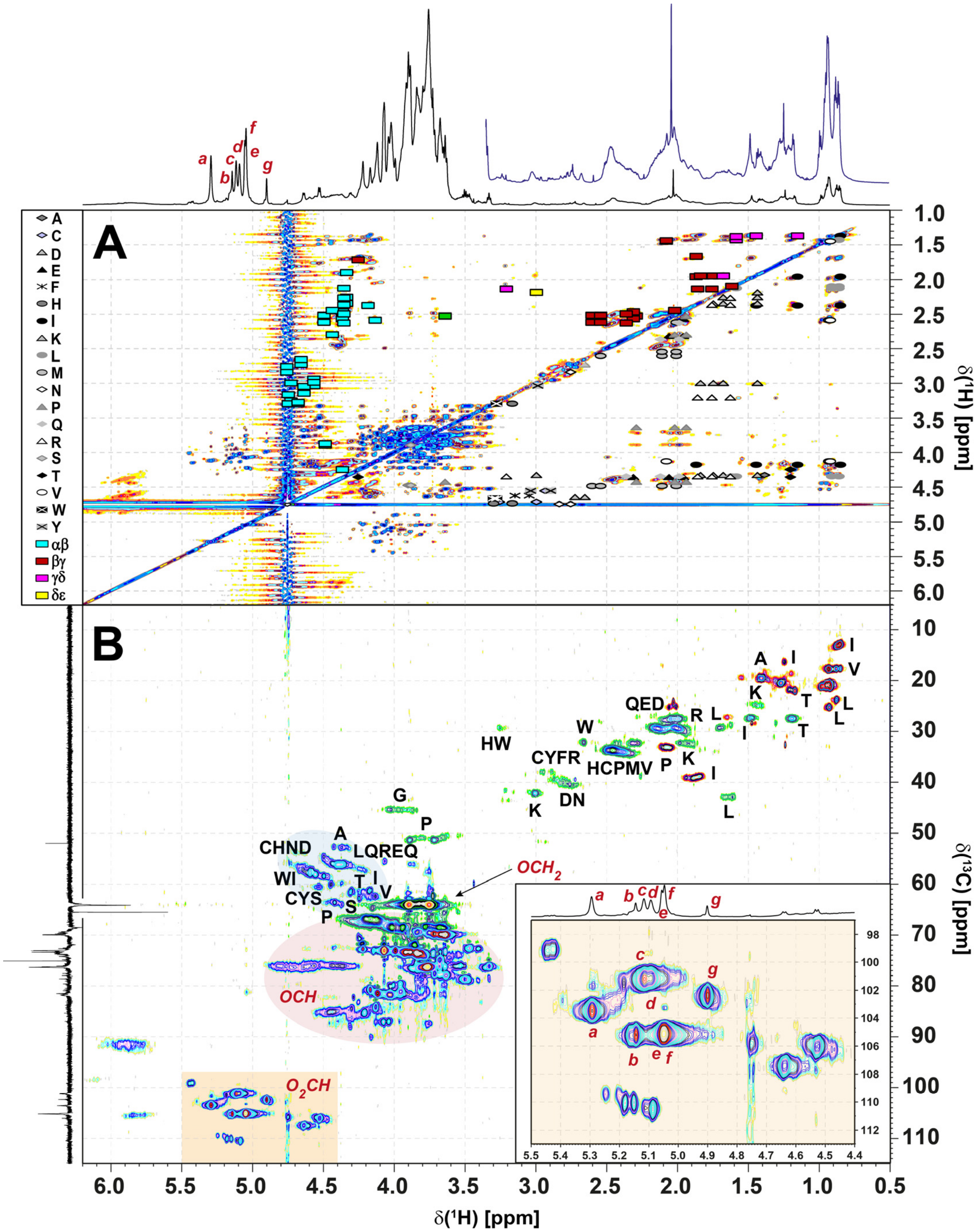

Fig. S1. (Panel A) ^{1}H, ^{1}H TOCSY and (panel B) ^{1}H, ^{13}C DEPT HSQC NMR spectra of EPS-2, aliphatic section, with cross peaks of proteinaceous amino acids (see attendant single letter code) in proteins following alanine (A) annotated according to position and carbon multiplicity (Hertkorn et al., 2002). (Panel A): upper left half: amino acid-derived COSY cross peaks according to positioning in peptides; (panel A): lower right half: amino acid-derived TOCSY cross peaks according to individual amino acids (individual gray symbols; single letter code for amino acids). (Panel B): ^{1}H, ^{13}C DEPT HSQC NMR spectrum, with ^{13}C DEPT-135 NMR projection spectrum in F1; color code: blue: CH; green: CH_2, red: CH_3. Orange shaded insert denotes section of anomeric $O_2\underline{CH}$ cross peaks, with major cross peaks indicated by letters a-g; other carbohydrate-related substructures are oxyomethylene $O\underline{CH_2}$, largely from carbohydrates and oxymethine $OC_2\underline{CH}$ ("*OCH*") groups.

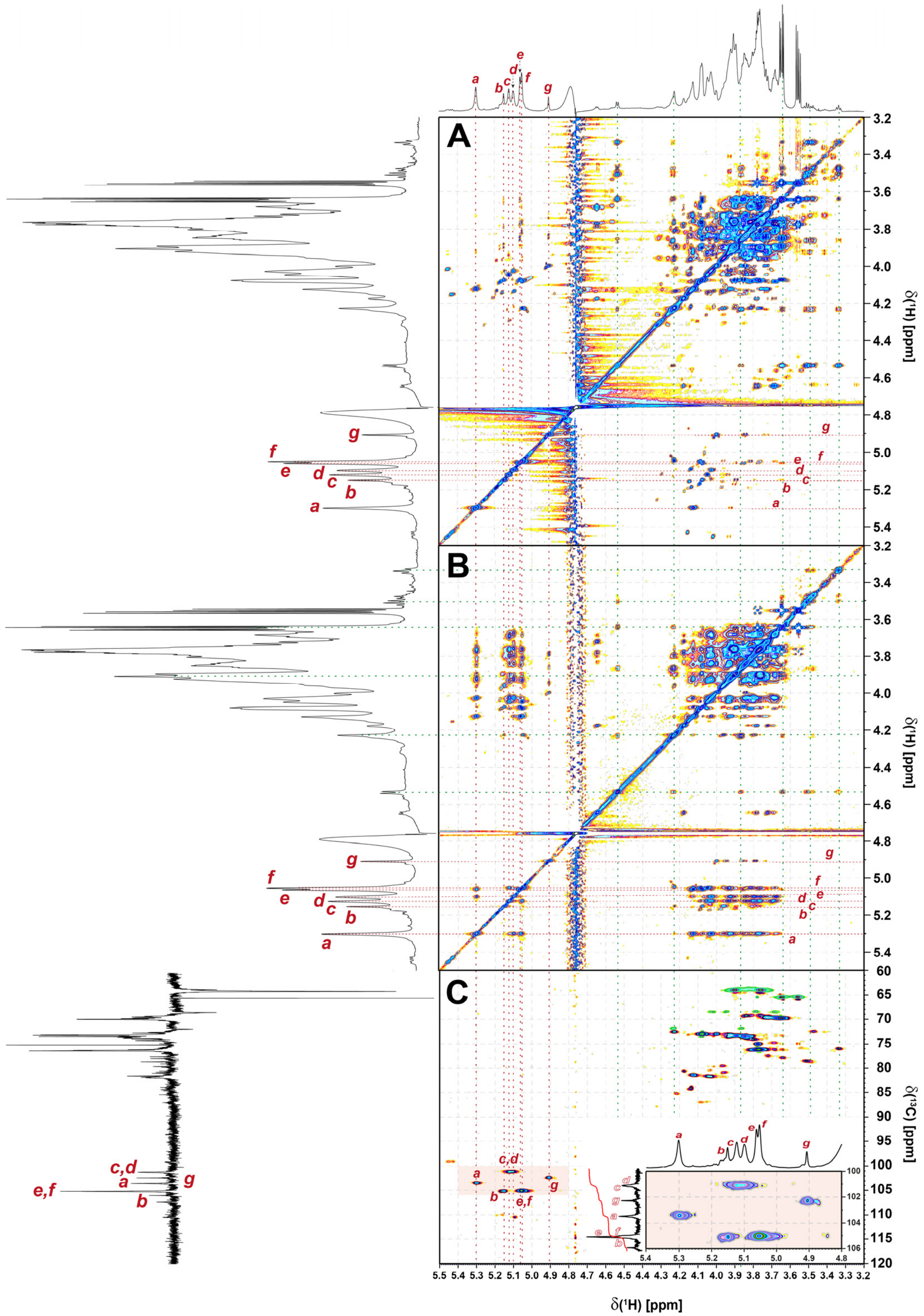

Fig. S2. (panel A) ^{1}H, ^{1}H NMR TOCSY NMR (panel B) ^{1}H, ^{1}H NMR NOESY NMR and (panel C) ^{1}H, ^{13}C DEPT HSQC NMR spectrum of EPS preparation BGI-2 (800 MHz, D_2O), with seven major anomeric NMR resonances of anomeric positions O_2CH, a-g, provided (cf. main text).

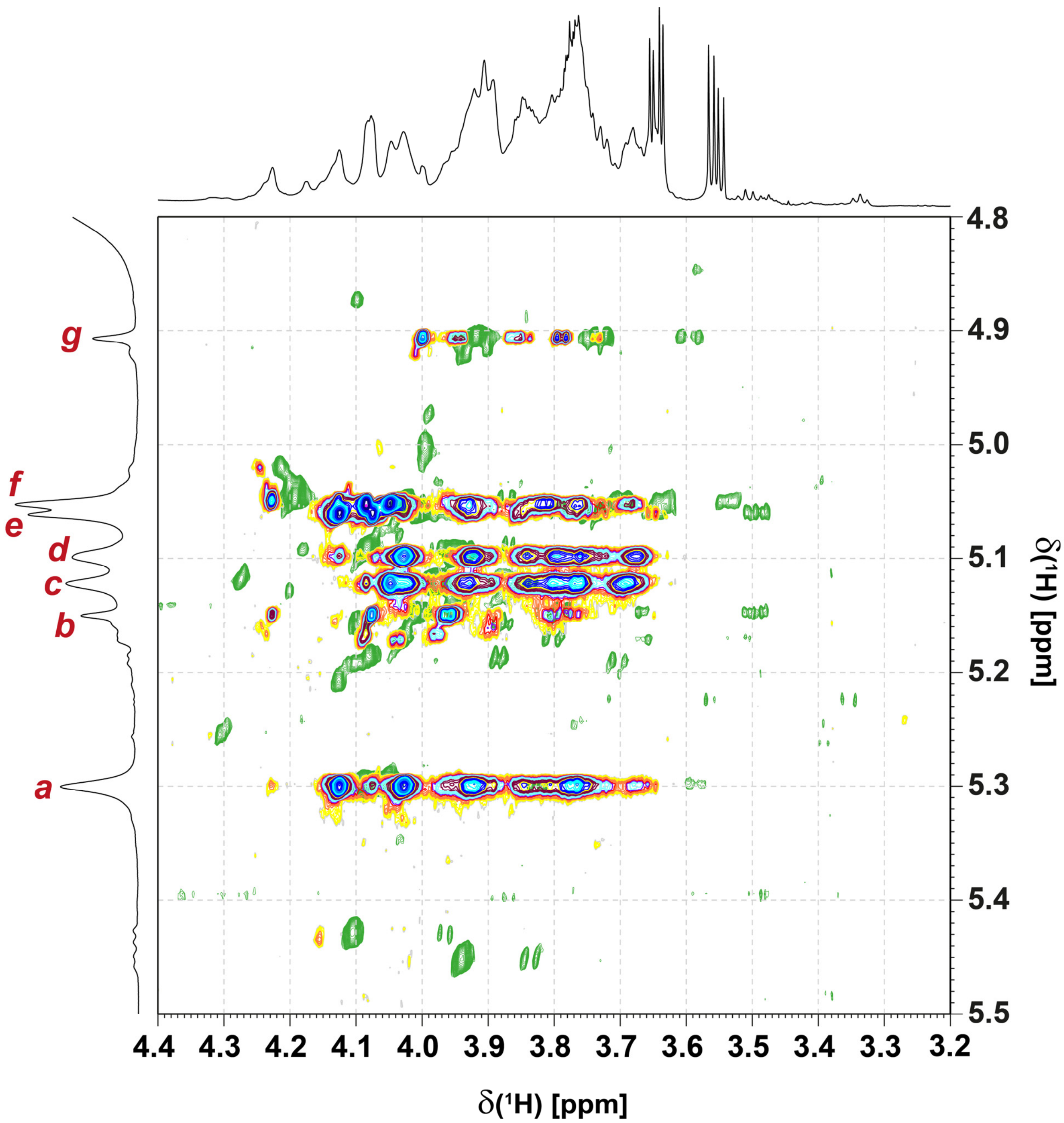

Fig. S3. Overlay of ^{1}H, ^{1}H TOCSY and (green color) ^{1}H, ^{1}H NOESY NMR spectra of EPS-2; section of anomeric O_2CH units (F1: with annotated seven major NMR resonances) versus section of OCH and OCH_2-units (F2); expansive cross peaks indicate superposition of closely related chemical environments.

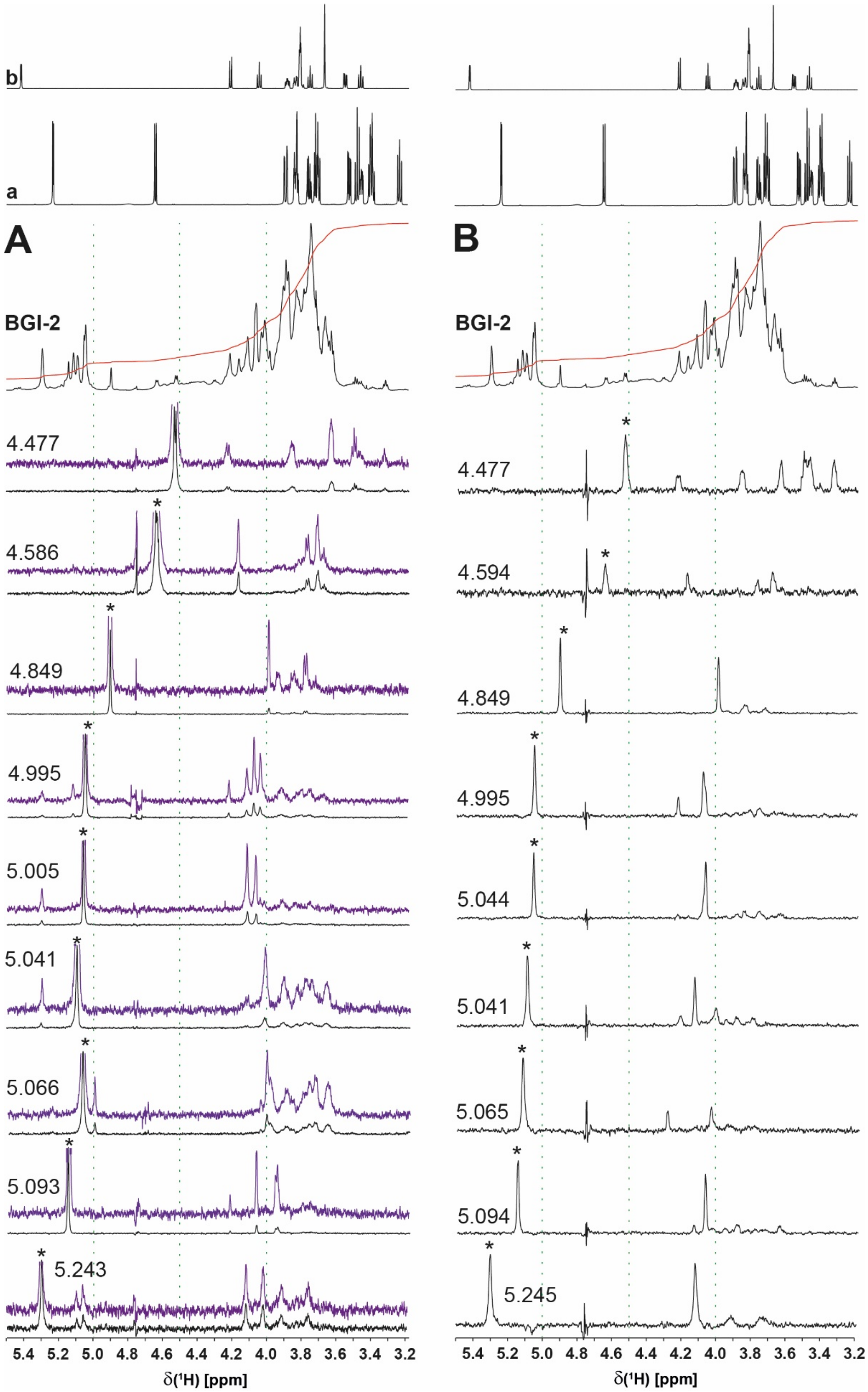

Fig. S4. Selective excitation of (panel A) 1D ^{1}H TOCSY and (panel B) 1D ^{1}H NOESY NMR experiments of BGI-2 with nominal δ_H of irradiation provided; top rows show standard ^{1}H NMR spectra of (a) D-glucose, (b) sucrose, BGI-2.
